# Global variances in infection control practices for vancomycin resistant *Enterococcus* – results of an electronic survey

**DOI:** 10.1186/s13756-016-0140-5

**Published:** 2016-11-03

**Authors:** H. Isenman, J. Michaels, D. Fisher

**Affiliations:** 1 Division of Infectious Diseases, National University Hospital, Singapore, Singapore; 2 Yong Loo Lin School of Medicine, National University of Singapore, Singapore, Singapore

**Keywords:** VRE, Infection control, International guidelines

## Abstract

An electronic survey was disseminated amongst infection control practitioners to investigate the variation in practices toward vancomycin resistant enterococci. There were 235 analysable responses, mostly from doctors, in 48 countries. The survey considered active surveillance, in-hospital precautions and tagging and untagging processes. There was a great variation in responses between and within countries highlighting a gap in guidance for practitioners on which to base institutional policy.

**Electronic supplementary material:**

The online version of this article (doi:10.1186/s13756-016-0140-5) contains supplementary material, which is available to authorized users.

## Introduction

Vancomycin resistant *Enterococcus* (VRE) emerged internationally through the 1990’s. Prevalences today vary widely, with between 0 and 45 % of *E. faecium* isolates demonstrating glycopeptide resistance in European countries [1]. In the United States of America (US) 3 % of hospital acquired infections were due to VRE [2]. The risk of invasive disease in VRE colonized patients is 4 % but can be upto 14 %, in immune suppressed patients, highlighting the importance of infection control measures especially in countries with a high prevalence [3, 4].

In the last 10 years, however, uncertainty has increased regarding the infection control prioritisation of this organism. Initially there were concerns over horizontal gene transfer and the possible emergence of Vancomycin Resistant *S. aureus* but this situation has not been realized. The treatment paradigm for VRE improved with the advent of linezolid and daptomycin in 2000 and 2003.

Now, in the era of carbapenemase producers and polymixin resistance, many question where as a priority VRE control should sit. The optimal approach to screening, isolation and surveillance is unclear, reflected by the paucity of international guidelines for VRE screening and infection control, with the most recent published in the United Kingdom and by the US Centres for Disease Control and Prevention (CDC) in 2006 [5, 6]. Inevitably, individual institutions have had to develop their own infection control approaches for VRE.

We sought to determine the approach to VRE control in different centres internationally.

## Methods

An electronic survey was created on survey monkey [7]. The survey was kept concise to facilitate contributions and required just a few minutes. The full survey is attached in the Additional file 1.

### Recruitment of participants

The link was forwarded to members of the International Society of Chemotherapy (ISC) infection control workgroup, the Infection Prevention Society (UK) and Oz bug (Australian list server). Personal contacts of the authors outside of these organisations were also invited to participate, and all recipients were asked to further refer the link to infection control practitioner colleagues world-wide.

**Fig. 1:**
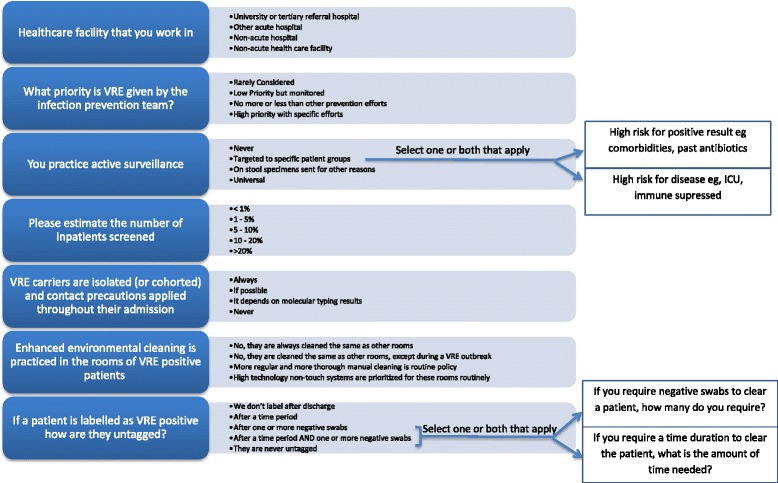
Non Demographic VRE Survey Questions (*arrows* show the follow on questions for respondents who selected the answer leading to the *arrow*)

Questions were multiple choice, most had a free text option and none were compulsory (Fig. 1). Optional demographic information included name, occupation, email address, type and country of institution. Areas assessed included VRE active surveillance, infection control precautions undertaken and labelling of patients. These terms were not specifically defined. Responses where the institution or country was not named were eliminated. Where more than one response was received from the same institution, the majority/most consistent response was selected and responses were merged.

The initial analysis reviewed all institutional responses, then a more focused analysis of countries with four or more institutions represented was performed to assess for international and intranational variation.

## Results

### Demographics

There was a total of 235 responses from 48 countries, with 189 institutions represented (see Table 1 for the full country list). Of the 234 who provided occupation details, 161 (69 %) were doctors, 51 (22 %) nurses, and 22 (9 %) other including medical laboratory scientists, pharmacists, infection control staff, clinical scientists microbiologists and an epidemiologist.

**Table 1:** Countries with four or more institutions represented: (Number, with percentage relating to the total responses from that country per specific question)

	Screening				Cleaning				Untagging				
Country (No Institutions Responded)	*Universal*	*Never*	*Targeted*	*On stool*	*Same as others*	*Increase in outbreak*	*High tech non touch*	*More than others*	*Don’t label*	*After time*	*After swab*	*After time + swab*	*Never Untag*
Australia (37)	3 (8)	3 (8)	31 (84)	-	2 (6)	6 (18)	1 (3)	25 (73)	2 (6)	3 (9)	3 (9)	16 (45)	11 (31)
Canada (4)	3 (75)	-	1 (25)	-	-	-	-	4 (100)	-	-	1 (25)	2 (50)	1 (25)
China (4)	-	1 (25)	1 (25)	2 (50)	1 (25)	-	-	3 (75)	-	1 (25)	2 (50)	1 (25)	-
Denmark (8)	4 (57)	1 (14)	2 (28)	-	-	1 (14)	3 (43)	3 (43)	-	2 (29)	3 (43)	1 (14)	1 (14)
Malaysia (6)	1 (17)	4 (66)	-	1 (17)	-	2 (33)	-	4 (67)	2 (33)	-	1 (17)	-	3 (50)
Netherlands (5)		1 (20)	4 (80)	-	1 (25)	-	-	4 (80)	-	-	-	5 (100)	-
New Zealand (7)	-	1 (14)	5 (72)	1 (14)	-	1 (17)	1 (17)	4 (66)	-	-	-	2 (33)	4 (67)
Singapore (7)	-	-	7 (100)	-	-	2 (40)	-	3 (60)	-	1 (14)	-	6 (86)	-
Turkey (5)	-	-	5 (100)	-	-	-	-	5 (100)	1 (20)	-	-	2 (40)	2 (40)
UK (31)	5 (17)	10 (33)	12 (40)	3 (10)	4 (14)	4 (14)	4 (14)	16 (57)	5 (18)	1 (3)	3 (11)	2 (7)	17 (21)
USA (22)	-	8 (36)	11 (50)	3 (14)	11 (50)	4 (20)	4 (20)	2 (10)	2 (9)	3 (14)	7 (32)	6 (27)	4 (18)

Countries with fewer than four institutional responses: Namibia, Argentina, Brazil, Belarus, Belgium, Bulgaria, Chile, DRC, Egypt, France, Germany, Greece, Hungary, Iceland, India, Japan, Malta, Nepal, Nigeria, Norway, Pakistan, Portugal, Qatar, Republic of Korea, Romania, Russia, Slovakia, Slovenia, South Africa, Spain, Sri Lanka, Sweden, Switzerland, Taiwan, Thailand

Of the 234 respondents who provided information regarding their place of work, the majority were in acute care, with 177 (76 %) from a tertiary referral centre, and 48 (20 %) from an acute care hospital. Only nine (4 %) were from a non-acute health care facility or hospital.

### Priority of VRE

VRE was given a high infection control priority with specific efforts in 95 (51 %) of the 187 institutions that responded to the question. It was prioritized “similar to other infection prevention efforts” by 51 (27 %), low priority 28 (15 %) and rarely considered in only 13 (7 %).

### Screening

Of 187 institutional responses to screening, active surveillance was practiced by 135 (72 %) with 18 (9 %) of those stating it was universal, and the remaining targeted. A minority, 39 (21 %) performed no surveillance at all, and 13 (7 %) performed surveillance on stool cultures sent for other reasons.

Of the 117 institutions that employ targeted screening, 42 (36 %) didn’t respond to the question regarding which target group they select. 52 (44 %) screen those that are at high risk for disease such as the immune suppressed, and 23 (20 %) targeted patients at high risk for a positive result such as previous antibiotic exposure or multiple comorbidities. Fourteen (12 %) selected both groups for screening.

Of 113 institutions quantifying the admissions screened for VRE, 94 (83 %) would screen up to 10 %; (25 (22 %) less than 1 %, 42 (37 %) 1–5 %, 5–10 %, 27 (24 %)). Only 19 (17 %) screened more inpatients, with 8 (7 %) screening 10–20 %, and 11 (10 %) screening over 20 % of inpatients.

### Type of contact precautions

Of the 178 who completed the question, 116 (65 %) always isolate identified colonized patients, 47 (26 %) isolate if possible, and 10 (5 %) never isolate: Namibia (1), Egypt (1), Sri Lanka(1), Argentina (1), DRC (1), Nigeria (1), USA (4). Only five (3 %) institutions use molecular typing results to guide isolation strategies: Australia (1), Canada (1), China (1), UK (3).

### Environmental cleaning

Of 171 who responded, 93 (54 %) perform more regular and more thorough manual cleaning. Fifty eight (34 %) would clean the same as other rooms, of which 28 (16 %) would use increased cleaning efforts during an outbreak. High technology non touch systems were employed by 20 (12 %) in UK (4), USA (4), Denmark (3), Belgium, (2), Malta, Nepal, Belarus, New Zealand, Romania, Qatar and Australia (1 each).

### ‘Untagging’ of colonized patients

Of 173 responding institutions, 54 (31 %) would not untag patients. Of the 96 (56 %) who do untag patients, 12 (7 %) would untag patients after a time period alone, 28 (16 %) would untag after a negative swab(s) alone, and 56 (33 %) require both a time period and one or more negative swabs to untag. Twenty three (13 %) do not label patients after discharge.

Of the 82 institutions that described their swab requirement for untagging patients, the majority of institutions require 2, (28 %) or 3, (54 %) swabs to be negative, whilst 10 % required one swab only. Seven respondents (8 %) required 4 or more swabs before untagging a patient as a VRE carrier.

The time period to clear patients likewise demonstrated great variation. Of the 68 institutions that nominated a time period as a criterion just 58 quantified it with 47 (81 %) clearing patients within a year: 15 (26 %) 12 months, 17 (29 %) 6 months, and 15 (26 %) 3 months. Only 10 (17 %) required 24 months to clear a patient, and 1 (1 %) over 24 months.

### Intranational variation

Eleven countries (Australia, Canada, China, Denmark, Malaysia, Netherlands, New Zealand, Singapore, Turkey, UK, US) had responses from four or more institutions. VRE was highly prioritized or prioritized the same as other MDROs by the majority of countries, however in the US there was almost equal spread between affording VRE a high priority (36 %), the same as other MDROs (27 %) and a low priority (36 %). The UK also varies in its approach with 43 % giving VRE high priority, 27 % the same as other MDROs and 23 % a low priority, with 7 % rarely considering it. Table 1 outlines the approaches to screening, cleaning and untagging.

There was less heterogeneity in the approach to contact precautions, with most countries always using contact precautions in over 50 % of institutions (with the exception of Turkey at 40 %), and the remainder of institutions employing contact precautions where possible. The USA showed a varied approach between institutions with universal use in 82 % and never using contact precautions in 18 %. In only Canada, Singapore and Turkey did all institutions responding undertake active surveillance. All other countries had respondents divided on the issue of active surveillance or not. Full data on contact precautions, VRE prioritization and number of inpatients screened can be found in the Additional file 2: Table S1.

## Discussion

This international survey has highlighted the variation in infection control approaches to VRE, within and between countries. Internationally, VRE still registers as an agent of concern with 81 % prioritizing it equal to or above other infection control efforts. Surveillance is undertaken in 72 % of responding institutions, with the majority rationalizing and finding merit in targeting high risk groups mostly by risk of severe disease, but many by risk of a positive result.

Although we received responses from 48 countries; we were reluctant to equate small numbers of responses from one country as truly representative; indeed the widest variation in approach was seen in countries with the greatest number of institutional responses such as the UK and US.

Practices also don’t necessarily reflect local epidemiology as VRE prevalence in the UK is 21 % [1], however it is seen as high priority in only 43 % of responding institutions and accordingly a third of institutions undertaken no active screening. Countries with a lower prevalence of VRE such as Denmark (4.5 %) and the Netherlands (1.1 %), prioritise VRE more highly (80 and 75 %), and more consistently isolate patients (71 and 100 %), alongside tagging at discharge [1].

The different approaches to infection prevention processes across institutions in the UK and USA, may in part be due to the fact that the infection control guidelines for these countries were last updated in 2006 [5, 6]. For instance active surveillance in the UK guideline is reserved for outbreaks while in the US there are several choices including point prevalence surveillance periodically, testing of “at risk” patients and testing roommates of VRE positive patients. Such advice now is not adhered to nor is it relevant in the setting of endemic VRE.

Responses from countries with more recently updated guidelines such as Singapore [8] showed greater yet still imperfect consistency.

The variance in approaches within any country may also be explained by differing views on the efficacy of contact precautions to prevent transmission. A study of discontinuation of contact precautions of haematology patient’s admitted to single rooms in a unit in a before/after study showed no significant difference in VRE blood stream infection (BSI) between time periods [9]. Another Canadian study comparing rates of VRE BSI between hospitals that employ screening and isolation and those that abandoned screening, found a rise in VRE BSI in all hospitals, but the rate of increase was highest in non-screening hospitals – this study is not yet complete however [10].

In terms of environmental cleaning, it was notable that 11 % use high technology non touch cleaning systems. The benefit of this technology has been shown to significantly reduce VRE acquisition in new occupants of rooms previously occupied by VRE positive patients [11].

Emerging as a “casual” survey, the response was beyond the authors’ expectations. We believe this was because the questions in hand were of genuine interest to respondents in these 48 countries. Furthermore, the study was designed to attract responses in being brief, and permitting a response from anyone with self perceived adequate local knowledge. No question was compulsory so survey respondents could progress if they could not or preferred no to answer, although still each question saw >100 responses. These strengths were also to some extent a weakness. Our study has many inherent shortcomings. There was no selection of institutions or participants and no submissions have been validated. Additionally, as we did not provide definitions for terms used such as untagging, it is possible that some questions were misinterpreted as it is hard to believe that universal active surveillance (intended to mean rectal swabbing and culture of patients on admission) is undertaken to this extent. The method of recruitment of participants provides selection bias towards more active infection control teams, and therefore more aggressive approaches may be overrepresented in the survey.

Despite the shortcomings of our methods, we found considerable variation in reported practices. Untagging of patients is either never done, or automatic on discharge or in between where the time course can be months to years and negative swabs required can be from 0 to 6. Given the lack of data in this area to inform practice, this variation is unsurprising. Practitioners in hospitals therefore are forced to create institutional policy. These policies and therefore practices will be based on personal viewpoints, local epidemiology (national as well as affecting that healthcare facility), the available infrastructure and capacity to isolate as well as competition for infection control resources from newer emerging multi drug resistant organisms (MDROs).

Our survey highlights reported variation in infection control practices related to VRE. Further research on optimal infection prevention practices and development of guidance in this area is warranted. The survey represents a request from infection control practitioners in 48 countries for the development of a guideline that can be adapted to a nation’s or a hospital’s requirements. Until that time practices will remain chaotic with the ensuing suboptimal use of infection control resources internationally.

## Additional files

Additional file 1: The full survey questionairre. (PDF 95 kb)
Additional file 2: Table S1. Countries with four or more institutions represented: (Number, with percentage relating to the total responses from that country per specific question). (DOCX 14 kb)

## Abbreviations

BSI: Blood stream infection
CDC: Centres for disease control and prevention
ISC: International society for chemotherapy
MDROs: Multi drug resistant organisms
UK: United Kingdom
US: United States (of America)
VRE: Vancomycin resistant Enterococci
