# Supplementary material for: Global variances in infection control practices for vancomycin resistant Enterococcus – results of an electronic survey
Source: Antimicrob Resist Infect Control. 2016 Nov 3;5:41. doi: 10.1186/s13756-016-0140-5 (PMC5094057; doi:10.1186/s13756-016-0140-5)
Supplement: Additional file 1: — The full survey questionairre. (PDF 95 kb) [file 13756_2016_140_MOESM1_ESM.pdf]

## International survey for VRE infection prevention

A quick scan to better understand the practices of VRE control internationally.

**Thank you for your participation. The survey will take less than 3 minutes to complete.**

1. Name

[ ]

2. Discipline

- ○ Doctor
- ○ Nurse
- ○ Other (please specify)

[ ]

* 3. What country do you work in?

[ ]

4. What type of health care facility do you work at?

- ○ University or tertiary referral hospital
- ○ Other acute hospital
- ○ Non-acute hospital
- ○ Non-acute health care facility

5. Please name the healthcare facility that you work in

[ ]

6. What priority is VRE given by the infection prevention team?

- ○ Rarely considered
- ○ Low priority but monitored
- ○ No more or less than most infection prevention efforts
- ○ High priority with specific efforts

7. You practice active surveillance

- Never
- On stool specimens sent for other reasons
- Targeted to specific patient groups
- Universal

## International survey for VRE infection prevention

8. As you selected 'target to specific patient groups' please select one or both that apply

- High risk for disease eg, ICU, immune supressed
- High risk for positive result eg comorbidities, past antibiotics

9. Please estimate the number of inpatients that are screened for VRE

- <1% of inpatients
- 1-5% of inpatients
- 5-10% of inpatients
- 10-20% of inpatients
- >20% of inaptinets

## International survey for VRE infection prevention

10. VRE carriers are isolated (or cohorted) and contact precautions applied throughout their admission

- Never
- If possible
- It depends on molecular typing results
- Always

11. Enhanced environmental cleaning is practiced in the rooms of VRE positive patients

- No, they are always cleaned the same as other rooms
- No, they are cleaned the same as other rooms, except during a VRE outbreak
- More regular and more thorough manual cleaning is routine policy
- High technology non-touch systems are prioritized for these rooms routinely

12. If a patient is labelled as VRE positive how are they untagged?

- We don't label after discharge
- After a time period
- After one or more negative swabs
- After a time period AND one or more negative swabs
- They are never untagged

## International survey for VRE infection prevention

13. If you require negative swabs to clear a patient, how many do you require?

- 1
- 2
- 3
- 4
- N/A
- >4, please state how many swabs

14. If you require a time duration to clear a patient, what is the amount of time needed?

- ◯ 3 months
- ◯ 6 months
- ◯ 12 months
- ◯ 24 months
- ◯ N/A
- ◯ >24, please state how many months
