# Supplementary material for: Global variances in infection control practices for vancomycin resistant Enterococcus – results of an electronic survey
Source: Antimicrob Resist Infect Control. 2016 Nov 3;5:41. doi: 10.1186/s13756-016-0140-5 (PMC5094057; doi:10.1186/s13756-016-0140-5)
Supplement: Additional file 2: Table S1. — Countries with four or more institutions represented: (Number, with percentage relating to the total responses from that country per specific question). (DOCX 14 kb) [file 13756_2016_140_MOESM2_ESM.docx]

**Supplementary table:**

**Countries with 4 or more institutions represented:**

**(Number, with percentage relating to the total responses from that country per specific question)**

|  | **VRE priority** | | | | **Contact Precautions** | | | | **% Inpatients Screened** | | | | |
| --- | --- | --- | --- | --- | --- | --- | --- | --- | --- | --- | --- | --- | --- |
| **Country**  (No. of institutions responded) | *High* | *Same as other MDRO* | *Low but monitored* | *Rarely consider* | *Always* | *Never* | *If possible* | *Mol typing* | *< 1%* | *1 – 5%* | *5 – 10%* | *10 – 20%* | *>20%* |
| **Australia (37)** | 21 (57) | 15 (40) | 1 (3) | - | 25 (69) | - | 10 (28) | 1 (3) | 1 (3) | 10 (33) | 13 (44) | 4 (13) | 2 (7) |
| **Canada (4)** | 4 (100) | - | - | - | 3 (75) | - | - | 1 (25) | - | - | - | - | 1 (100) |
| **China (4)** | 2 (50) | - | 2 (50) | - | 2 (50) |  | 1 (25) | 1 (25) | - | 1 (100) | - | - | - |
| **Denmark (8)** | 6 (75) | 2 (25) | - | - | 5 (71) | - | 2 (29) | - | 1 (50) | - | - | - | 1 (50) |
| **Malaysia (6)** | 1 (17) | 5 (83) | - | - | 4 (67) | - | 2 (33) | - | - | - | - | - | - |
| **Netherlands (5)** | 4 (80) | 1 (20) | - | - | 5 (100) | - | - | - | 1 (25) | 2 (50) | 1 (25) | - | - |
| **New Zealand (7)** | 4 (66) | 1 (17) | - | 1 (17) | 6 (100) | - | - | - | 2 (40) | 3 (60) | - | - | - |
| **Singapore (7)** | 3 (43) | 3 (43) | 1 (14) | - | 5 (71) | - | 2 (29) | - | 2 (29) | 4 (57) | 1 (14) | - | - |
| **Turkey (5)** | 4 (80) | 1 (20) | - | - | 2 (40) | - | 3 (60) | - | 1 (20) | 3 (60) |  |  | 1(20) |
| **UK (31)** | 13 (43) | 8 (27) | 7 (23) | 2 (7) | 14 (50) | - | 12 (43) | 2 (7) | 5 (45) | 3 (27) | 2 (18) | 1 (9) | - |
| **USA (22)** | 8 (36) | 6 (27) | 8 (36) | - | 18 (82) | 4 (18) | - | - | 3 (28) | 4 (36) | - | 2 (18) | 2 (18) |
|  | | | | | | | | | | | | | |
